# Supplementary material for: Oligomerization mediated by the D2 domain of DTX3L is critical for DTX3L‐PARP9 reading function of mono‐ADP‐ribosylated androgen receptor
Source: Protein Sci. 2024 Mar 21;33(4):e4945. doi: 10.1002/pro.4945 (PMC10955461; doi:10.1002/pro.4945)
Supplement: Supplementary file 1 — DATA S1. Supporting information. [file PRO-33-e4945-s001.pdf]

Oligomerisation mediated by the D2 domain of DTX3L is critical for DTX3L-PARP9 reading function of mono-ADP-ribosylated androgen receptor

Carlos Vela-Rodríguez[1], Chunsong Yang[2], Heli I. Alanen[1], Rebeka Eki[3], Tarek A. Abbas[3], Mirko M. Maksimainen[1], Tuomo Glumoff[1], Ramona Duman[4], Armin Wagner[4], Bryce M. Paschal[2,*] & Lari Lehtiö[1,*]

[1]Faculty of Biochemistry and Molecular Medicine & Biocenter Oulu, University of Oulu, Finland.

[2]Department of Biochemistry and Molecular Genetics, University of Virginia, USA

[3]Department of Radiation Oncology, University of Virginia, USA

[4]Diamond Light Source, Harwell Science and Innovation Campus, Didcot OX11 0DE, UK.

*Corresponding authors: bmp2h@virginia.edu or lari.lehtio@oulu.fi

Contents

Figure S1. Representative electron density map.

Figure S2. Superimposition of D2 structure with AlphaFold model of DTX3L.

Table S1. Results obtained from the DALI search against the PDB25 dataset and comparison with FoldSeek search against PDB100.

Table S2. Results obtained from the Foldseek search against the PDB100 dataset above 15% probability and their comparison with the Dali search against PDB25.

Table S3. Results obtained from the Foldseek search against the AlphaFold database above 30% probability.

Table S4. Structural parameters of the SAXS data of the D2 domain obtained from the data analysis with ScÅtter.

Figure S3. Point-specific amino acid conservation of the D2 domain of DTX3L

Figure S4. Recombinant protein inputs related to Figure 4.

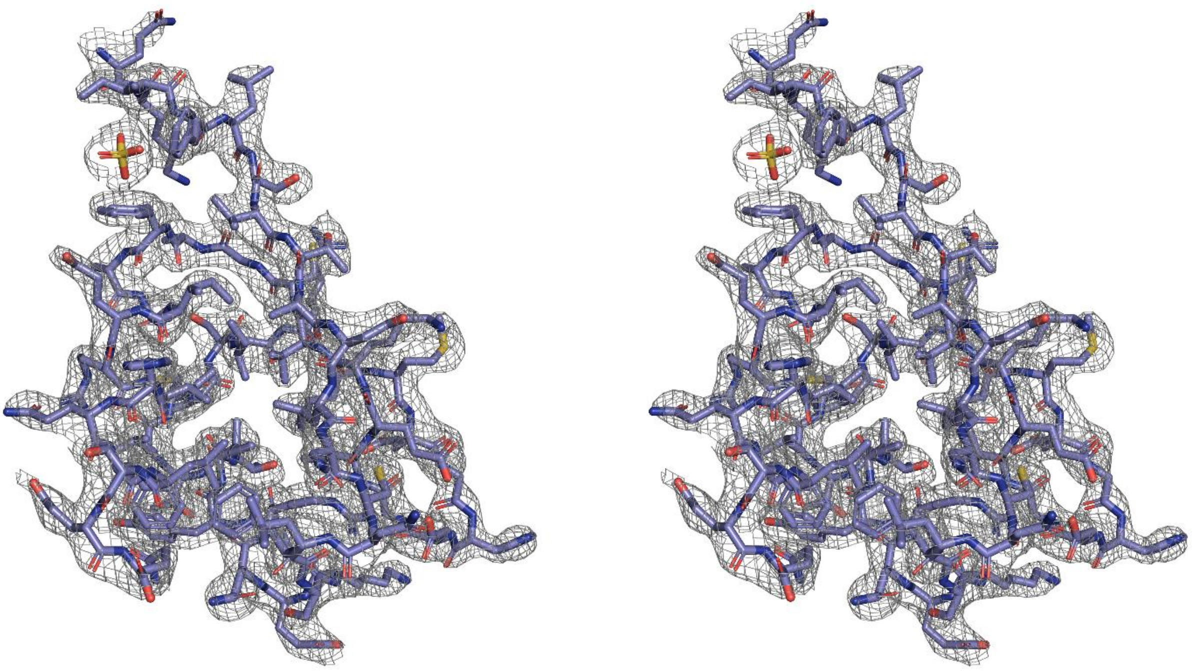

Figure S1. Stereoscopic representation of the electron density map of a monomer of the D2 domain of DTX3L. The electron density map is contoured at 1.0 sigma.

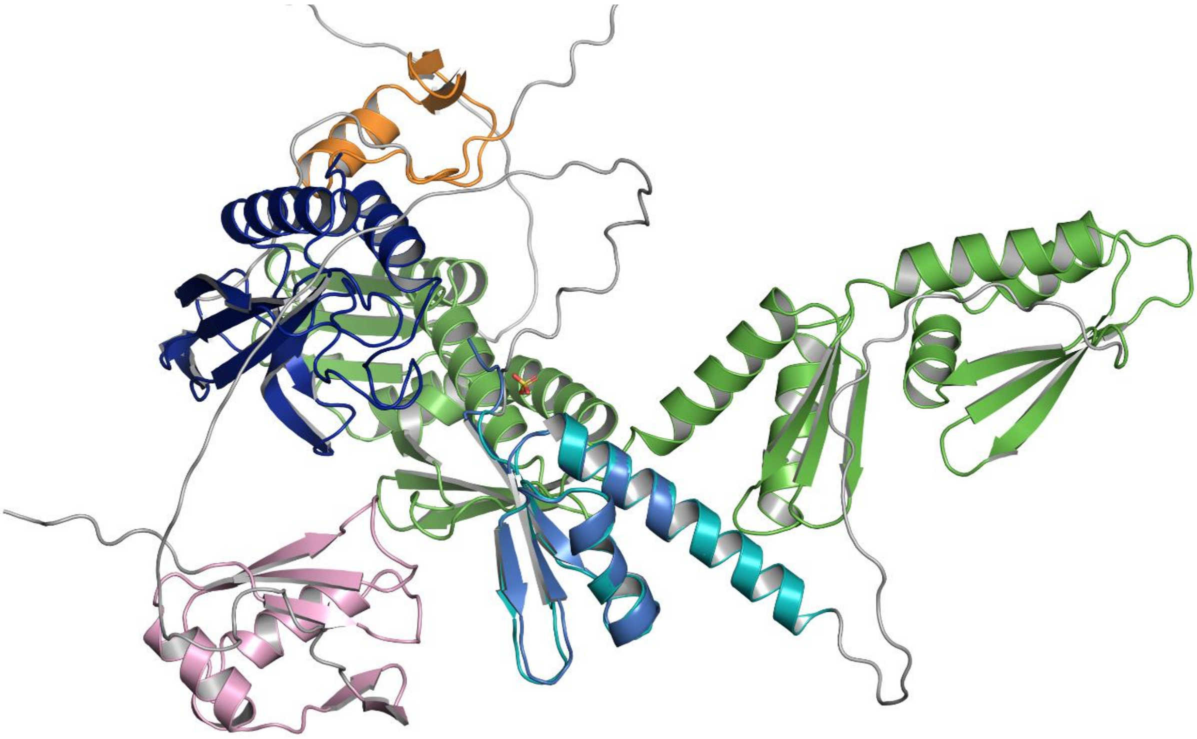

Figure S2. Superimposition of a monomer of the experimentally solved structure (marine) to the predicted full-length structure of DTX3L. Domains of DTX3L are colour-coded as the schematic in **Figure 1A**. AlphaFold2 prediction shows long, flexible regions between the domains of the protein.

Table S1. Results obtained from the DALI search against the PDB25 dataset and comparison with FoldSeek search against PDB100.

Chain	Z score	RMSD	Aligned residues	% id	PDB Description	Identified by Foldseek (Probability score out of 1.00; E-value)
5zdh-A	5.6	3.7	57	2	TYPE II SECRETION SYSTEM PROTEIN D	Yes (0.08; 5.07e+0)
7eqx-A	5.4	2.7	56	11	CARBOXYPEPTIDASE B	No
4ec5-A	5.3	3.6	58	14	GENERAL SECRETION PATHWAY PROTEIN D	No
5hvf-A	5.1	2.6	54	9	CARBOXYPEPTIDASE B2	No
6w6m-A	5.1	3.0	55	9	TYPE IV PILUS SECRETIN PILQ FAMILY PROTEIN	No
4av2-A	5.1	2.5	58	9	TYPE IV PILUS BIOGENESIS AND COMPETENCE PROTEIN P	No
6n10-A	5.0	3.4	57	9	DIPHOSPHOMEVALONATE DECARBOXYLASE MVD1. PEROXISOM	No
8h6s-A	4.9	3.1	58	9	MALONYL-COA-[ACYL-CARRIER-PROTEIN] TRANSACYLASE	No
6u45-A	4.9	3.3	55	11	ELONGATION FACTOR 2	No
6wrv-C	4.9	2.1	50	8	TRANSFERRIN RECEPTOR PROTEIN 1	Yes (0.14; 1.77e+0)
6wi5-A	4.9	1.9	51	12	DE NOVO DESIGNED PROTEIN FOLDIT4	No
6wrw-C	4.9	2.2	49	8	TRANSFERRIN RECEPTOR PROTEIN 1	Yes (0.10; 3.1e+0)
3ce8-A	4.8	2.4	52	8	PUTATIVE PII-LIKE NITROGEN REGULATORY PROTEIN	No
1jqg-A	4.7	2.6	52	12	CARBOXYPEPTIDASE A	No
6fij-A	4.7	3.1	54	9	POLYKETIDE SYNTHASE	No
2n2u-A	4.6	2.1	51	12	OR358	Yes (0.06; 7.19e+0)
4qbu-A	4.6	3.0	52	17	ZMAA	No
1i7q-A	4.6	2.6	51	8	ANTHRANILATE SYNTHASE	No
5oqj-2	4.6	3.5	54	4	DNA-DIRECTED RNA POLYMERASE II SUBUNIT RPB1	No
1pyt-A	4.6	2.8	55	13	PROCARBOXYPEPTIDASE A	No
1in0-A	4.5	2.5	50	10	YAJQ PROTEIN	Yes (0.06; 3.33e+0)
6qum-N	4.4	3.4	56	7	V-TYPE ATP SYNTHASE ALPHA CHAIN	No
4gx2-B	4.3	2.9	53	9	TRKA DOMAIN PROTEIN	No
3cj8-A	4.3	2.4	49	16	2.3.4.5-TETRAHYDROPYRIDINE-2.6-DICARBOXYLATE N-	No
5d4o-A	4.3	2.9	53	4	NITROGEN REGULATORY PROTEIN P-II	No
7lmx-A	4.3	2.5	55	9	INTEGRIN INHIBITOR	No
7cyf-D	4.3	2.8	52	8	SLR1512 PROTEIN	No
2pfd-A	4.3	2.8	55	7	FORMIMIDOYLTRANSFERASE-CYCLODEAMINASE	No
3f56-B	4.2	2.3	51	4	CSOS1D	No
5wx8-A	4.2	2.8	56	2	IMMEDIATE-EARLY PROTEIN 2	No
1wey-A	4.1	3.1	55	4	CALCIPRESSIN 1	Yes (0.02; 5.07e+0)
1sb6-A	4.1	2.4	50	8	COPPER CHAPERONE SCATX1	Yes (0.18; 1.65e+0)

3s1e-A	4.1	2.4	52	6	CYTOKININ DEHYDROGENASE 1	No
2g9o-A	4.1	2.3	50	12	COPPER-TRANSPORTING ATPASE 1	No
3v2u-C	4.1	2.8	54	11	GALACTOSE/LACTOSE METABOLISM REGULATORY PROTEIN G	No
7t71-A	4.0	4.3	53	6	MEVALONATE 3.5-BISPHOSPHATE DECARBOXYLASE	No
8csp-8	4.0	2.9	53	6	28S RIBOSOMAL PROTEIN S34. MITOCHONDRIAL	No
6fon-A	4.0	2.8	51	8	COPPER CHAPERONE FOR SUPEROXIDE DISMUTASE	No
6jsh-B	4.0	2.9	55	11	FATTY ACID SYNTHASE SUBUNIT BETA	No
2l48-A	4.0	3.2	57	5	N-ACETYLMURAMOYL-L-ALANINE AMIDASE	No
1ec6-A	4.0	2.2	51	12	20-MER RNA HAIRPIN	No
7np8-B	4.0	3.5	54	15	COENZYME F420-DEPENDENT SULFITE REDUCTASE	No
4u9r-A	4.0	3.4	55	16	CZCP CATION EFFLUX P1-ATPASE	No
4zck-A	4.0	2.6	53	17	GTP-BINDING PROTEIN TYPA/BIPA	No
2wbm-A	4.0	3.0	54	17	RIBOSOME MATURATION PROTEIN SDO1 HOMOLOG	No
3v97-A	3.9	2.4	48	8	RIBOSOMAL RNA LARGE SUBUNIT METHYLTRANSFERASE L	No
7uvp-A	3.9	3.4	55	9	TETRACYCLINE RESISTANCE PROTEIN TETQ	No
8hcn-D	3.9	2.9	55	9	UREASE SUBUNIT GAMMA	No
2k1r-B	3.9	2.6	49	6	COPPER-TRANSPORTING ATPASE 1	Yes (0.04; 4.40e+0)
3mah-A	3.9	3.3	53	13	ASPARTOKINASE	No
8gh6-A	3.9	3.4	53	8	REVERSE TRANSCRIPTASE-LIKE PROTEIN	No
6eml-p	3.9	3.5	57	9	PRE-18S RIBOSOMAL RNA	No
7nad-U	3.9	2.5	50	8	25S RRNA	No
3oq2-B	3.8	2.6	51	6	CRISPR-ASSOCIATED PROTEIN CAS2	Yes (0.10; 1.54e+0)
7agv-F	3.8	3.5	52	2	K(+)/H(+) ANTIPORTER SUBUNIT KHTT	No
6cc2-A	3.8	3.4	54	7	CELL DIVISION CONTROL PROTEIN 45 CDC45 PUTATIVE	No
5wwx-A	3.8	2.9	52	10	RNA-BINDING E3 UBIQUITIN-PROTEIN LIGASE MEX3C	Yes (0.23; 6.18e+0)
2ywg-A	3.8	3.7	57	14	GTP-BINDING PROTEIN LEPA	No
5www-A	3.8	2.5	54	11	RNA-BINDING E3 UBIQUITIN-PROTEIN LIGASE MEX3C	No
6yaq-A	3.8	1.9	49	4	CYTOKININ DEHYDROGENASE 8	No
5cwa-A	3.8	4.1	58	7	ANTHRANILATE SYNTHASE COMPONENT 1	No
3kiz-B	3.8	2.5	52	8	PHOSPHORIBOSYLFORMYLGLYCINAMIDINE CYCLO-LIGASE	No
1eqr-A	3.8	3.4	55	5	ASPARTYL-TRNA SYNTHETASE	No
5gan-G	3.7	3.0	55	7	SACCHAROMYCES CEREVISIAE STRAIN UOA_M2 CHROMOSOME	No
4ct8-A	3.7	3.6	57	14	CINA-LIKE PROTEIN	No

8d8j-F	3.7	2.5	51	14	PROBABLE S-ADENOSYL-L-METHIONINE-DEPENDENT RNA	No
7ock-L	3.7	2.1	51	6	S-ADENOSYLMETHIONINE SYNTHASE	No
7qri-A	3.7	2.9	56	9	TRYPTOPHAN 5-HYDROXYLASE 2	No
1ahu-A	3.7	3.0	55	5	VANILLYL-ALCOHOL OXIDASE	No
1q5y-D	3.6	2.7	52	8	NICKEL RESPONSIVE REGULATOR	No
6eld-A	3.6	3.0	52	6	NUCLEOLYSIN TIA-1 ISOFORM P40.U1 SMALL NUCLEAR	No
6vej-A	3.6	3.5	53	8	PROBABLE RESISTANCE-NODULATION-CELL DIVISION (RND	No
5yys-A	3.6	3.3	54	7	L-FUCOKINASE. L-FUCOSE-1-P GUANYLYLTRANSFERASE	No
4yut-A	3.6	3.3	54	6	FAMILY 3 ADENYLATE CYCLASE	No
6l5d-B	3.6	3.1	52	6	GAS VESICLE PROTEIN	No
2pff-B	3.6	2.9	54	11	FATTY ACID SYNTHASE SUBUNIT ALPHA	No
6fht-B	3.5	3.8	55	5	BACTERIOPHYTOCHROME.ADENYLATE CYCLASE	No
6zvp-A	3.5	2.3	52	4	TYROSINE 3-MONOOXYGENASE	No
1f93-A	3.5	2.2	53	8	DIMERIZATION COFACTOR OF HEPATOCYTE NUCLEAR	No
7nhr-A	3.5	3.2	53	15	PUTATIVE TRANSMEMBRANE PROTEIN WZC	No
6gwj-B	3.5	2.6	52	8	EKC/KEOPS COMPLEX SUBUNIT LAGE3	No
1v8c-A	3.5	2.9	50	14	MOAD RELATED PROTEIN	No
1q8l-A	3.5	2.5	52	8	COPPER-TRANSPORTING ATPASE 1	No
3ui3-B	3.4	2.3	50	8	IMMUNOGLOBULIN G-BINDING PROTEIN G. VIRULENCE-ASS	No
3c6k-D	3.4	2.2	50	8	SPERMINE SYNTHASE	No
4qmf-B	3.4	3.5	55	5	KRR1 SMALL SUBUNIT PROCESSOME COMPONENT	No
6nx5-A	3.4	3.6	51	6	PUMILIO DOMAIN-CONTAINING PROTEIN C56F2.08C	No
6bwo-A	3.4	2.8	49	8	PYRIDINIUM-3.5-BISTHIOCARBOXYLIC ACID MONONUCLEOT	No
4pwu-C	3.4	3.0	53	8	MODULATOR PROTEIN MZRA	No
7qh2-C	3.4	3.2	55	7	LACTATE DEHYDROGENASE (NAD(+).FERREDOXIN) SUBUNIT	No
6j6g-C	3.4	3.4	55	7	PRE-MRNA-SPLICING FACTOR 8	No
6s6b-K	3.4	2.4	52	2	CRISPR-ASSOCIATED PROTEIN. CMR5 FAMILY	No
3afg-B	3.4	3.2	56	13	SUBTILISIN-LIKE SERINE PROTEASE	No
4lir-B	3.4	3.3	52	6	NUCLEOPORIN NUP53	No
6teq-A	3.4	3.0	55	7	GALACTOKINASE	No
2jvz-A	3.4	2.4	52	8	FAR UPSTREAM ELEMENT-BINDING PROTEIN 2	No
7m7h-B	3.4	3.4	52	10	ERYAI.6-DEOXYERYTHRONOLIDE-B SYNTHASE ERYA3. MODU	No

4aim-A	3.4	3.9	58	12	POLYRIBONUCLEOTIDE NUCLEOTIDYLTRANSFERASE	No
1u8s-B	3.4	3.0	53	2	GLYCINE CLEAVAGE SYSTEM TRANSCRIPTIONAL	No
6pwn-A	3.3	3.9	56	11	SMALL-CONDUCTANCE MECHANOSENSITIVE CHANNEL	No
3dkx-A	3.3	3.3	55	15	REPLICATION PROTEIN REPB	No
7wvz-A	3.3	3.5	51	10	BETA-KETOACYL-ACYL-CARRIER-PROTEIN SYNTHASE I	No
1fd8-A	3.3	2.7	48	8	ATX1 COPPER CHAPERONE	No
2ko1-A	3.3	2.8	53	8	GTP PYROPHOSPHOKINASE	No
4zos-A	3.3	3.1	54	2	PROTEIN YE0340 FROM YERSINIA ENTEROCOLITICA SUBSP	No
6mrj-B	3.3	2.8	53	11	NICKEL-RESPONSIVE REGULATOR	No
4olp-B	3.3	2.4	49	4	GRPU MICROCOMPARTMENT SHELL PROTEIN	No
2gx8-C	3.3	2.4	53	4	NIF3-RELATED PROTEIN	No
7v99-A	3.3	3.3	53	4	TELOMERASE REVERSE TRANSCRIPTASE	No
6dgd-A	3.3	2.7	53	8	PRIMOSOMAL PROTEIN N'	No
3wx4-A	3.3	3.5	54	9	ANTI-RESTRICTION ENDONUCLEASE	No
1yqh-A	3.3	2.5	52	2	IG HYPOTHETICAL 16092	Yes (0.08; 1.08e+0)
1vr6-A	3.3	3.0	50	4	PHOSPHO-2-DEHYDRO-3-DEOXYHEPTONATE ALDOLASE	No
3gnw-B	3.3	3.4	51	6	RNA-DIRECTED RNA POLYMERASE	No
1zav-A	3.2	2.0	50	8	50S RIBOSOMAL PROTEIN L10	No
2hfs-A	3.2	3.8	53	9	MEVALONATE KINASE. PUTATIVE	No
4zoq-F	3.2	2.6	47	13	INTRACELLULAR SERINE PROTEASE	No
7qpr-D	3.2	3.3	49	12	ACT DOMAIN PROTEIN	No
6s2e-A	3.2	3.7	56	11	DNA POLYMERASE EPSILON CATALYTIC SUBUNIT A	No
2wbr-A	3.2	3.6	53	8	GW182	No
6pwj-A	3.2	2.6	54	11	GGDEF AND EAL DOMAIN-CONTAINING PROTEIN	No
7jtk-i	3.2	2.8	48	6	FLAGELLAR RADIAL SPOKE PROTEIN 1	No
7cv0-A	3.2	3.8	50	6	TRANSCRIPTIONAL REGULATOR NIAR	No
2f3j-A	3.2	3.8	50	6	RNA AND EXPORT FACTOR BINDING PROTEIN 2	No
5u9m-D	3.2	2.4	46	9	SUPEROXIDE DISMUTASE [CU-ZN]	No
7q4l-A	3.2	5.0	55	7	DEAD END PROTEIN HOMOLOG 1	No
1kn6-A	3.2	2.9	52	6	PROHORMONE CONVERTASE 1	No
2hh2-A	3.1	2.5	48	4	KH-TYPE SPLICING REGULATORY PROTEIN	No
5hb7-A	3.1	3.2	53	8	NUCLEOPORIN NUP53	No
7nhr-C	3.1	3.5	54	15	PUTATIVE TRANSMEMBRANE PROTEIN WZC	No
4gzk-A	3.1	2.7	50	12	RNA-DEPENDENT RNA POLYMERASE P2	No
6wb2-A	3.1	3.3	52	13	HIV-1 VIRAL RNA GENOME FRAGMENT	No

3opk-C	3.1	3.3	56	4	DIVALENT-CATION TOLERANCE PROTEIN CUTA	No
6lpn-B	3.1	3.6	54	13	D-2-HYDROXYGLUTARATE DEHYDROGENASE. MITOCHONDRIAL	No
7m1n-A	3.1	2.9	47	9	PUTATIVE FERREDOXIN	No
3tvi-D	3.1	4.0	55	15	ASPARTOKINASE	Yes (0.03; 6.71e+0)
1qfr-A	3.1	2.6	51	8	PHOSPHOCARRIER PROTEIN HPR	No
5uyy-A	3.1	4.5	54	15	PREPHENATE DEHYDROGENASE	No
4usj-C	3.1	3.1	53	0	ACETYLGLUTAMATE KINASE. CHLOROPLASTIC	No
8ba1-A	3.1	3.7	54	6	CLEAVAGE AND POLYADENYLATION SPECIFICITY FACTOR S	No
8ily-A	3.1	3.8	53	6	SET DOMAIN CONTAINING 1A. HISTONE LYSINE METHYLTR	No
3j6v-J	3.1	3.0	55	7	28S RIBOSOMAL RNA. MITOCHONDIAL	No
2raq-B	3.1	2.8	53	9	CONSERVED PROTEIN MTH889	No
3ihs-A	3.1	3.3	52	8	PHOSPHOCARRIER PROTEIN HPR	No
1fx2-A	3.1	3.1	55	7	RECEPTOR-TYPE ADENYLATE CYCLASE GRESAG 4.1	No
7bbb-A	3.1	3.3	50	8	ATP-DEPENDENT RNA HELICASE DBPA	No
4wd9-A	3.1	2.9	53	11	NISIN BIOSYNTHESIS PROTEIN NISB	No
2ril-A	3.1	2.6	49	12	ANTIBIOTIC BIOSYNTHESIS MONOOXYGENASE	No
6me0-C	3.1	4.2	51	14	T.EL4H RNA	No
2lvw-A	3.1	3.8	55	5	ACETOLACTATE SYNTHASE ISOZYME 1 SMALL SUBUNIT	No
1yg0-A	3.1	2.3	45	11	COP ASSOCIATED PROTEIN	No
6cng-A	3.0	2.7	41	10	FATTY ACID KINASE (FAK) B3 PROTEIN	No
1siz-A	3.0	2.2	44	9	FERREDOXIN	No
8a8k-A	3.0	3.1	55	4	PAP PHOSPHATASE FROM METHANOTHERMOCOCCUS	No
6ner-E	3.0	2.8	52	6	BMC-H TANDEM FUSION PROTEIN	No
4p52-A	3.0	4.1	58	7	HOMOSERINE KINASE	No
3mcs-B	3.0	3.0	52	4	PUTATIVE MONOOXYGENASE	No
5anb-K	3.0	3.0	52	10	60S RIBOSOMAL PROTEIN L3	No
4dnr-A	3.0	2.5	53	9	CATION EFFLUX SYSTEM PROTEIN CUSB	No
1xpp-D	3.0	3.1	52	13	DNA-DIRECTED RNA POLYMERASE SUBUNIT L	No
3ced-A	3.0	3.6	55	13	METHIONINE IMPORT ATP-BINDING PROTEIN METN 2	No
6lxg-A	3.0	2.6	47	2	GTP PYROPHOSPHOKINASE	No
1fjg-F	3.0	2.5	50	14	16S RIBOSOMAL RNA	No
6dd5-A	3.0	3.7	53	6	MMB-1 CAS6 FUSED TO MALTOSE BINDING PROTEIN.CRISP	No
4v1a-k	3.0	2.5	53	4	MITORIBOSOMAL PROTEIN ML37. MRPL37	No
7r65-A	3.0	3.4	53	6	ADENYLATE/GUANYLATE CYCLASE	No

6k2e-A	3.0	2.6	50	4	CRISPR/CAS2 PROTEIN	No
3d45-A	3.0	3.8	53	11	POLY(A)-SPECIFIC RIBONUCLEASE PARN	No
5hy3-A	3.0	2.0	47	15	MRNA ENDORIBONUCLEASE LSOA	No
5tl4-A	3.0	2.9	53	4	VANILLATE/3-O-METHYLGALLATE O-DEMETHYLASE	No

Table S2. Results obtained from the Foldseek search against the PDB100 dataset above 15% probability and their comparison with the Dali search against PDB25.

Chain	Probability	E-value	Target alignment range (Query)	% id	PDB Description	Identified by DALI (Z score)
3jc8-Qa	0.38	1.54e+0	354-410 (6-59)	12.2	Architectural model of the type IVa pilus machine	No
1zzj-B	0.35	3.78e-1	6-71 (5-59)	16.6	Structure of the third KH domain of hnRNP K in complex with 15-mer ssDNA	No
3vke-A	0.35	4.67e-1	3-66 (5-59)	14	Contribution of the first K-homology domain of poly(C)-binding protein 1 to its affinity and specificity for C-rich oligonucleotides	No
5suh-B	0.30	1.43e+0	135-192 (6-59)	8.6	The structure of double ringed trimeric shell protein MSM0271 from the RMM microcompartment	No
3hjv-B	0.28	9.42e-1	132-184 (8-59)	14.2	Crystal structure of an acyl carrier protein S-malonyltransferase from Vibrio cholerae	No
5wq7-A	0.25	2.51e+0	4-62 (6-59)	8.4	CryoEM structure of type II secretion system secretin GspD	No
6i1x-B	0.25	2.18e+0	4-58 (6-55)	12.7	Aeromonas hydrophila ExeD	No
5wwz-A	0.23	6.18e-1	15-76 (5-59)	10.9	KH2 domain of human RNA-binding E3 ubiquitin-protein ligase MEX-3C	No
5wwx-A	0.23	6.18e-1	16-77 (6-59)	14.2	KH2 domain of human RNA-binding E3 ubiquitin-protein ligase MEX-3C complex with RNA	Yes (3.8)
4f3q-A	0.23	1.08+0	129-179 (6-59)	18.1	Structure of a YebC family protein (CBU_1566) from Coxiella burnetii	No
3u1k-B	0.23	8.78e-1	555-615 (5-59)	8	Crystal structure of human PNPase	No
1j5k-A	0.21	5.01e-1	1-70 (1-59)	15	COMPLEX OF THE KH3 DOMAIN OF HNRNP K WITH A SINGLE_STRANDED 10MER DNA OLIGONUCLEOTIDE	No
1u2r-A	0.21	8.18e-1	471-528 (6-60)	17.2	Crystal Structure of ADP-ribosylated Ribosomal Translocase from Saccharomyces cerevisiae	No
6smd-C	0.2	1.43e+0	131-184 (6-59)	8.6	PlMCAT:AntF (holo): type II PKS acyl-carrier protein in complex with its malonyl-transacylase	No
1sb6-A	0.18	1.65e+0	1-58 (5-59)	5.1	Solution structure of a cyanobacterial copper metallochaperone, ScAtx1	Yes (4.1)
5suh	0.18	1.77e+0	136-193 (6-59)	6.8	The structure of double ringed trimeric shell protein MSM0271 from the RMM microcompartment	No
6ve4-A	0.18	1.25e+0	3-57 (6-59)	8.3	Pentadecameric PilQ from Pseudomonas aeruginosa	No
3h0p-A	0.18	1.43e+0	131-184 (6-59)	8.6	Crystal Structure of an Acyl Carrier Protein S-malonyltransferase from Salmonella typhimurium	No
2p8z	0.18	1.08e+0	471-528 (6-60)	17.2	Fitted structure of ADPR-eEF2 in the 80S:ADPR-eEF2:GDPNP:sordarin cryo-EM reconstruction	No
2npf-B	0.16	1.34e+0	466-523 (6-60)	17.2	Structure of eEF2 in complex with moriniafungin	No
2p8w-T	0.16	1.16e+0	471-528 (6-60)	17.2	Fitted structure of eEF2 in the 80S:eEF2:GDPNP cryo-EM reconstruction	No

7qpx-B	0.15	1.43e+0	4-59 (6-60)	10.3	Complex of rice blast (Magnaporthe oryzae) effector protein AVR-PikC with an engineered HMA domain of Pikp-1 (Pikp-SNK-EKE) from rice (Oryza sativa)	No
4olo-C	0.15	2.51e+0	22-73 (6-59)	7.4	Ligand-free structure of the GrpU microcompartment shell protein from Clostridiales bacterium 1_7_47FAA	No
3ezj-E	0.15	5.43e+0	78-136 (6-59)	13.5	Crystal structure of the N-terminal domain of the secretin GspD from ETEC determined with the assistance of a nanobody	No
7nvv-2	0.15	3.33e+0	232-289 (1-60)	6.6	XPB-containing part of TFIIH in a post-translocated state (with ADP-BeF3)	No
3b78-E	0.15	1.90e+0	472-529 (6-60)	18.9	Structure of the eEF2-ExoA(R551H)-NAD+ complex	No
7ls2-M	0.15	1.08e+0	500-557 (6-60)	15.2	80S ribosome from mouse bound to eEF2 (Class I)	No

Table S3. Results obtained from the Foldseek search against the AlphaFold database above 30% probability.

Target	Probability	E-value	Target alignment range (Query)	% id	Organism	PDB Description
AF-Q8TDB6-F1-model_v4	1.00	1.67e-10	132-191 (1-60)	100	Homo sapiens	E3 ubiquitin-protein ligase DTX3L
AF-D3Z8X6-F1-model_v4	1.00	9.10e-9	145-202 (3-60)	68.9	Rattus norvegicus	E3 ubiquitin-protein ligase DTX3L
AF-Q3UIR3-F1-model_v4	1.00	7.91e-9	144-204 (1-60)	73.7	Mus musculus	E3 ubiquitin-protein ligase DTX3L
AF-A2BGH5-F1-model_v4	0.99	3.69e-2	144-204 (1-59)	21.3	Danio rerio	E3 ubiquitin-protein ligase DTX3L
AF-A0A0R4ITG3-F1-model_v4	0.66	3.24e-1	329-389 (6-60)	19.3	Danio rerio	Si:dkey-154b15.1
AF-A0A0H3GNK3-F1-model_v4	0.63	1.40e-1	15-70 (6-59)	22.4	Klebsiella pneumoniae subsp. pneumoniae HS11286	Uncharacterised protein
AF-A0A2K6WM31-F1-model_v4	0.60	3.73e-1	384-444 (6-58)	17.4	Onchocerca volvulus	Uncharacterised protein
AF-A0A0G2KFZ1-F1-model_v4	0.57	4.00e-1	162-219 (5-59)	10.3	Danio rerio	Poly [ADP-ribose] polymerase
AF-A0A0G2KLA5-F1-model_v4	0.57	4.60e-1	244-301 (5-59)	13.7	Danio rerio	Poly [ADP-ribose] polymerase
AF-Q57921-F1-model_v4	0.54	4.60e-1	6-59 (6-59)	18.1	Methanocaldococcus jannaschii DSM 2661	Uncharacterised protein
AF-M0R9K1-F1-model_v4	0.54	2.82e-1	388-453 (5-59)	16.6	Rattus norvegicus	Heterogeneous nuclear ribonucleoprotein K
AF-K0F977-F1-model_v4	0.54	1.07e+0	496-553 (1-59)	17.7	Nocardia brasiliensis ATCC 700358	Polyketide synthase
AF-Q9I319-F1-model_v4	0.51	8.06e-1	105-161 (6-59)	11.8	Pseudomonas aeruginosa PAO1	Type 3 secretion system secretin
AF-D4A8D6-F1-model_v4	0.44	5.29e-1	245-316 (1-59)	16.6	Rattus norvegicus	Uncharacterised protein
AF-A0A0N4UFX5-F1-model_v4	0.44	7.01e-1	383-444 (6-59)	17.1	Dracunculus medinensis	Uncharacterised protein
AF-A0A0G2KAA1-F1-model_v4	0.41	4.60e-1	124-186 (5-59)	9.0	Rattus norvegicus	RING-type domain-containing protein

AF-Q8IJS7-F1-model_v4	0.41	4.29e-1	731-789 (6-59)	18.3	Plasmodium falciparum 3D7	PRE-biding protein
AF-A0A0K0EIM2-F1-model_v4	0.35	6.09e-1	265-321 (6-57)	15.7	Strongyloides stercoralis	SAM domain-containing protein
AF-P35672-F1-model_v4	0.33	1.74e+0	107-165 (6-59)	8.4	Salmonella enterica subsp. enterica serovar Typhimurium str. LT2	SPI-1 type 3 secretion system secretin
AF-A0A3P7DCM4-F1-model_v4	0.33	6.09e-1	365-424 (5-58)	16.3	Wuchereria bancrofti	Uncharacterised protein
AF-P61980-F1-model_v4	0.3	3.73	388-453 (5-59)	15.9	Rattus norvegicus	Heterogeneous nuclear ribonucleoprotein K
AF-Q17832-F1-model_v4	0.30	9.95e-1	348-409 (6-59)	12.3	Caenorhabditis elegans	ViGiLN homologue
AF-E7FCA5-F1-model_v4	0.30	4.93e-1	362-427 (5-59)	16.4	Danio rerio	High density lipoprotein-binding protein b

Table S4. Structural parameters of the SAXS data of the D2 domain obtained from the data analysis with ScÅtter.

	Reciprocal space (Guinier analysis)	Real space [P(r) distribution]
I(0) (cm^{-1})	0.12 ± 0.0001	0.13 ± 0.05
Rg (Å)	28.7 ± 0.1	28.5 ± 19.2
Volume ($Å^3$)	103387	110720
d_{max}	-	112
Porod exponent	3.5 ± 0.1	
X^2 (total estimate)	-	0.78
Mass (kDa)	-	43

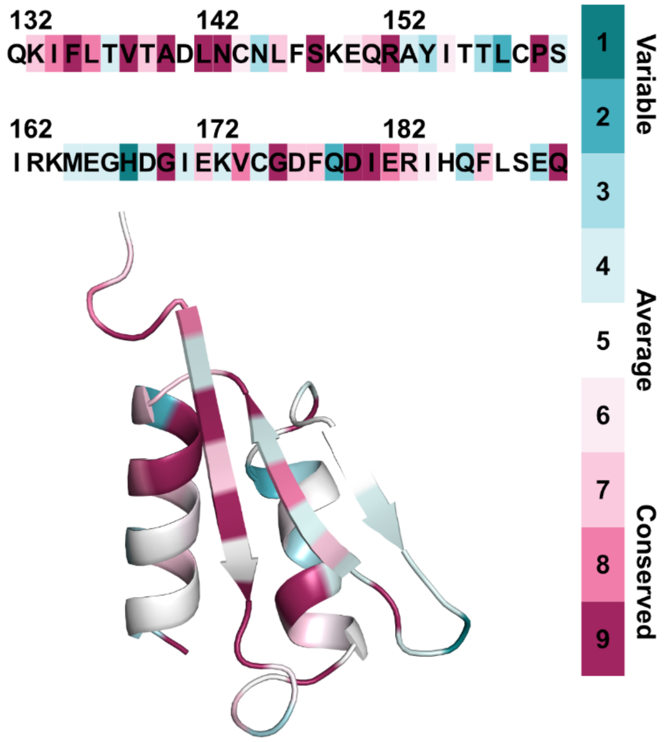

Figure S3. Point-specific amino acid conservation of the D2 domain of DTX3L determined by the ConSurf server using the chain A of the determined structure as an input. The cartoon representation is coloured based on the conservation scale shown on the right and the corresponding amino acid residues of the chain are shown on the top.

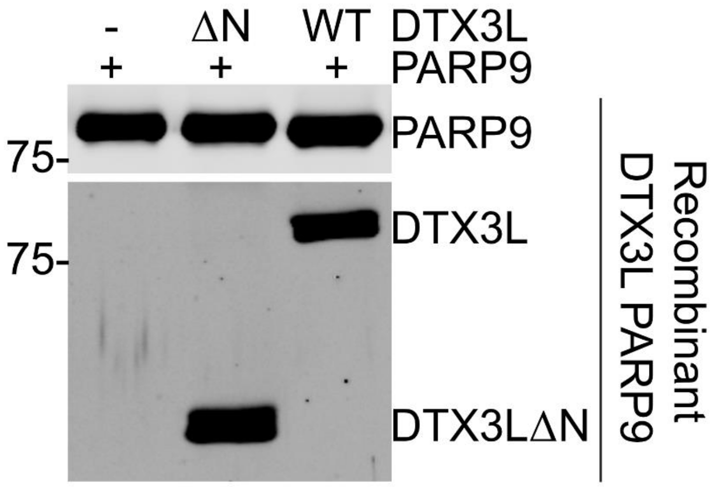

Figure S4. Recombinant protein inputs related to Fig. 4. Recombinant PARP9 alone or combined with DTX3L or DTX3LΔN (10 µM for each protein) was pre-incubated on ice for 6 h, and then diluted to 1 µM each with an extraction buffer [20 mM Tris-HCl (pH 7.5), 100 mM NaCl, 0.5% Triton X-100, 1 mM PMSF, 2 mM DTT, 5 mM EDTA, 5 µg/mL each of aprotinin/leupeptin/pepstatin with veliparib]. Equal volume of protein solution was further diluted with 1x SDS loading buffer and subjected to SDS-PAGE and Western blot analysis.
